# Supplementary material for: Modelling white matter in gyral blades as a continuous vector field
Source: Neuroimage. Author manuscript; Available in PMC 2021 May 17. (PMC7610793; doi:10.1016/j.neuroimage.2020.117693)
Supplement: Supplementary Material [file EMS123952-supplement-Supplementary_Material.docx]

7 Supplementary


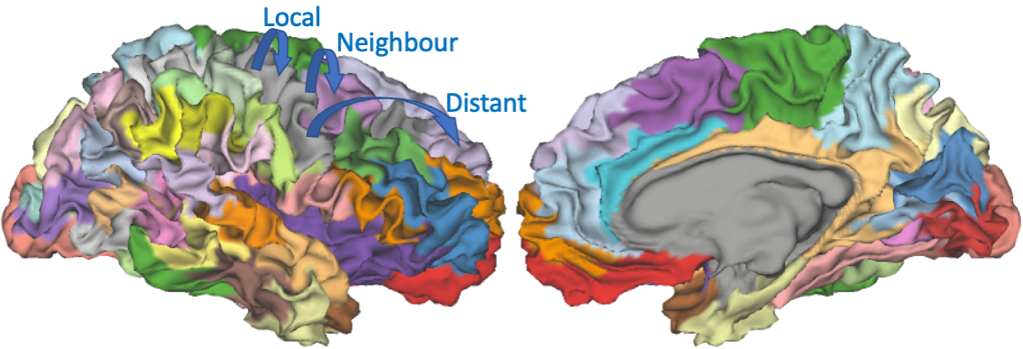


Figure S1 Definition of the intrahemispheric connections excluding local connections and U-fibres used in Figure 11. The cortex is sub-divided into 50 parcels (illustrated for single subject) with the borders between the parcels preferentially located in the sulcal fundi. Local connections and U-fibres are excluded by only considering the connectivity between vertices in different parcels that do not border each other. The parcellation is obtained using a watershed algorithm: the vertices are sorted by sulcal depth (from high to low) and then iterated through. Each vertex is assigned to a new parcel if none of its neighbours are in existing parcels (i.e., it is a local maximum) and otherwise assigned to the neighbouring parcels. If two parcels touch, they are merged if one of them is insufficient deep (i.e., maximum – minimum sulcal depth is below some threshold), otherwise they are kept separate. The depth threshold is chosen, so that we end up with 50 parcels.


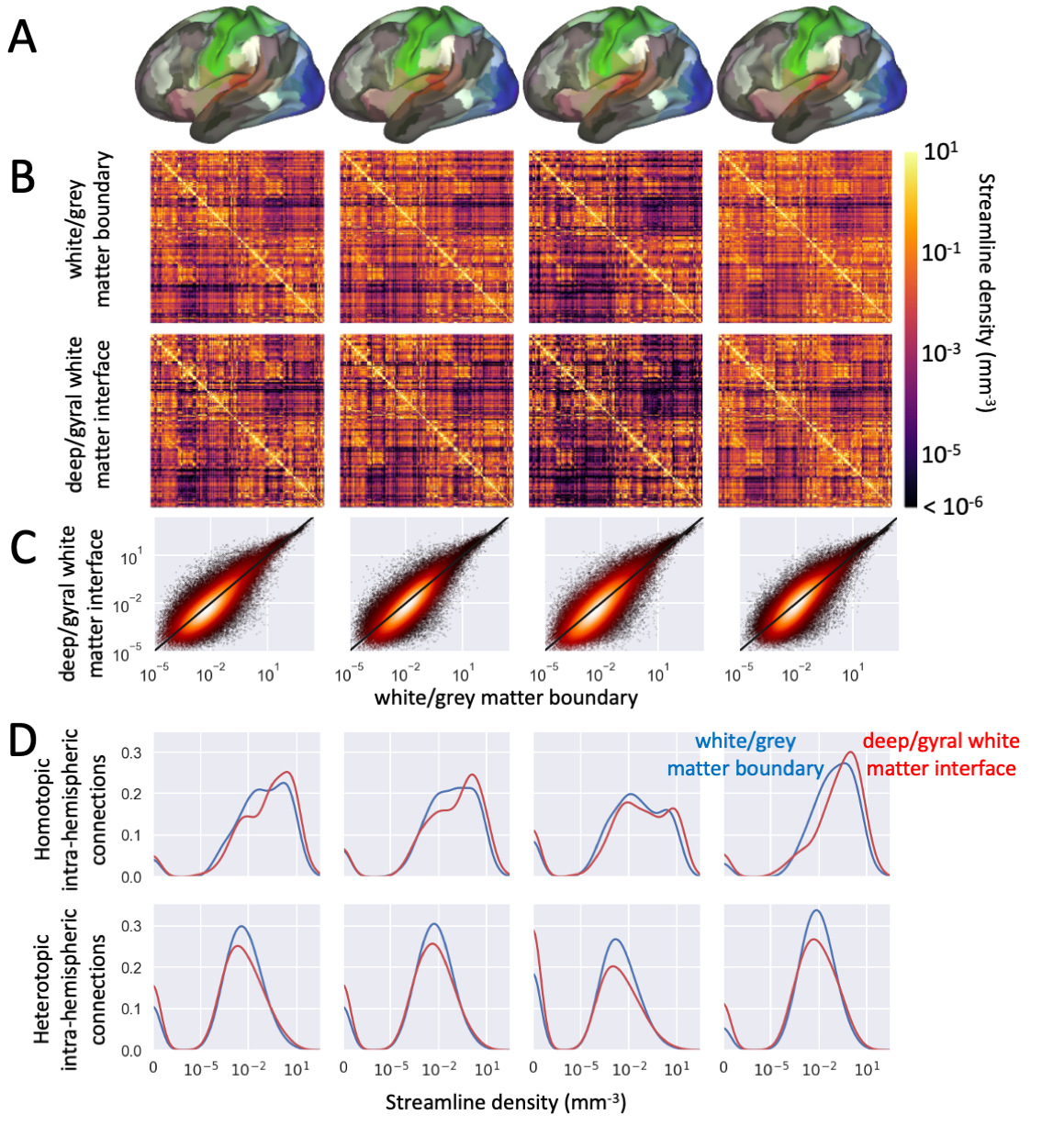


Figure S2 Analysis of parcellated connectomes for 4 subjects (one column per subject). A) Single-subject multi-modal parcellation from Glasser et al. (2016) used to parcellate the connectome. B) Heat map of the streamline density obtained using the white/grey-matter boundary (top) or the deep/gyral white matter interface. The parcellated connnectomes are very similar as also seen in the scatter plot (C). D) Distribution of streamline density for the interhemispheric streamline density between homotopic parcels (top) and heterotopic parcels (bottom). The homotopic connectivity has a median increase of 66%, 37%, 35%, and 53% for these 4 subjects when adopting the deep/gyral white matter interface (red), while the heterotopic connectivity has a median decrease of 10%, 11%, 8%, and 20%. The streamline density measures the number of streamlines seeded from the vertices in one parcel that terminate in each mm-3 of the target parcel.


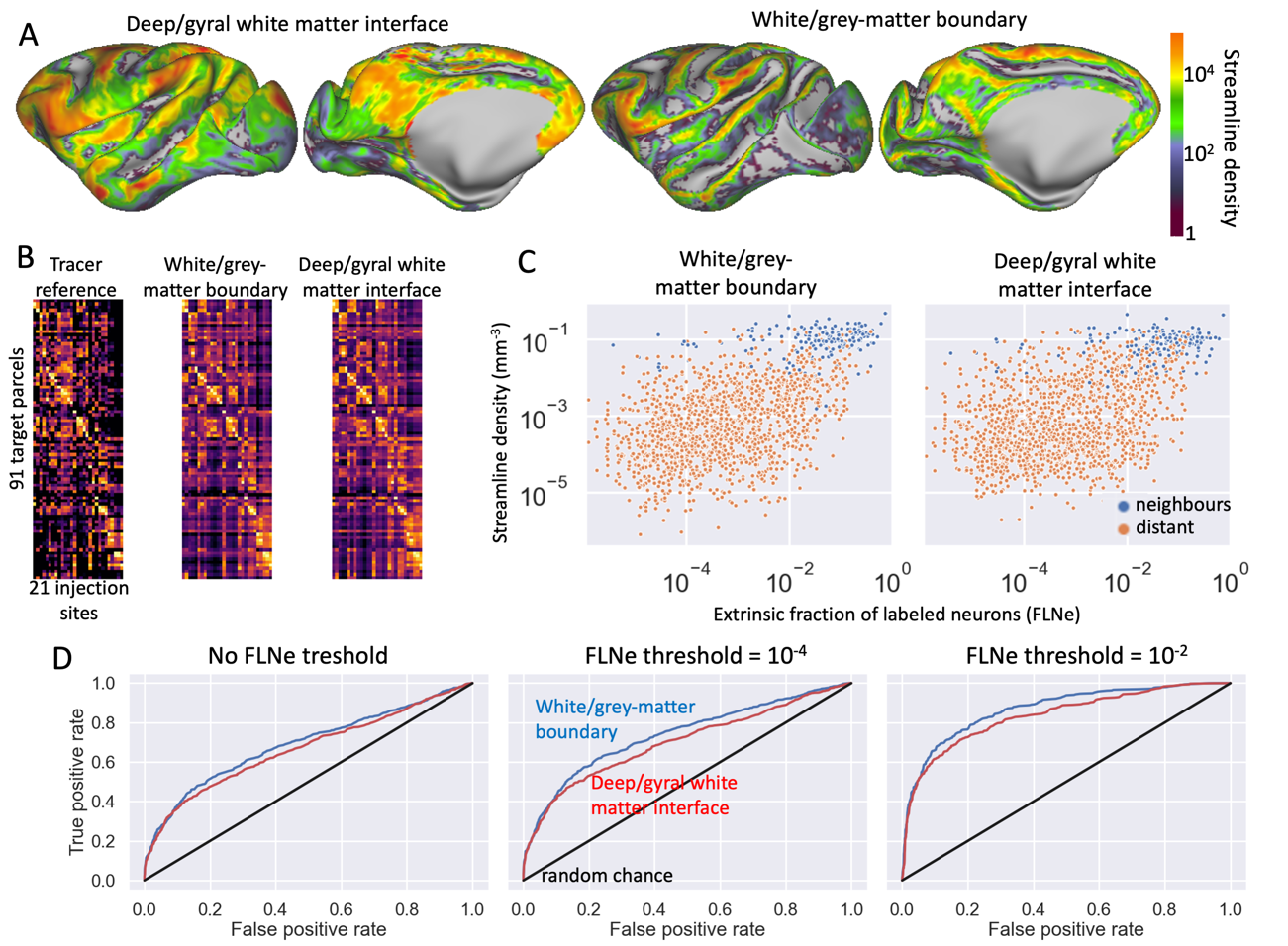


Figure S3 Comparison of the parcellated connectome of a macaque with tracer data from Markov et al. (2011; 2014). A) Illustration of the gyral bias in the macaque diffusion MRI data showing the density of interhemispheric streamline termination points (colour map is the same as in Figure 8A). B) Heatmap of the reference connectome with the log-transformed extrinsic fraction of labelled neurons (FLNe) on the left and the connectomes when using the white/grey-matter boundary in the middle and the deep/gyral white matter interface on the right. C) Correlation between the connectomes from tractography and the tracer connectome (only for the 62% of connections with non-zero connectivity). When adopting the deep/gyral white matter interface the correlation with the tracer connectome becomes worse. This negative trend becomes statistically insignificant when regressing out distance as in Donahue et al. (2016). D) ROC curves for predicting the “true” connections by thresholding the tractography connectomes for from left to right different thresholds of the FLNe. Again, the ROC curves are worse when adopting the deep/gyral white matter interface.
